# Exploring Disorders of Gut–Brain Interaction in Schoolchildren and Adolescents with Autism

**DOI:** 10.3390/life15060912

**Published:** 2025-06-04

**Authors:** Carlos Alberto Velasco-Benítez, Christian Andrés Rojas-Cerón, Claudia Jimena Ortiz-Rivera, Daniela Alejandra Velasco-Suárez, María Carolina Juvinao-Quintero, Cecilia Elena Zubiri, Julián Martín Fernández, Román Bigliardi, Anabella Zosi, Ricardo A. Chanis Águila, Celina Guzmán Acevedo, Fátima Azereth Reynoso Zarzosa, Roberto Arturo Zablah Cordova

**Affiliations:** 1 Departamento de Pediatría, Escuela de Medicina, Facultad de Salud, Universidad del Valle, Cali 76001, Colombia; carlos.velasco@correounivalle.edu.co (C.A.V.-B.); christian.andres.rojas@correounivalle.edu.co (C.A.R.-C.); 2 Faculty of Health Sciences, Universidad Libre, Cali 760031, Colombia; claudia.ortiz@correounivalle.edu.co; 3 Gastrohnup Research Group, Universidad del Valle, Hospital Universitario del Valle, Cali 76001, Colombia; velasco.daniela@correounivalle.edu.co; 4 Hospital de Niños Sor María Ludovica de La Plata, Buenos Aires 1900, Argentina; cecilia.zubiri.ar@findersgroup.org (C.E.Z.); anabella.zosi.ar@findersgroup.org (A.Z.); 5 Hospital Materno Infantil Dr. Florencio Escardó de Tigre, Buenos Aires 1648, Argentina; julian.fernandez.ar@findersgroup.org; 6 Hospital Nacional Profesor Alejandro Posadas, Buenos Aires 1684, Argentina; roman.bigliardi.ar@findersgroup.org; 7 Hospital del Niño Dr. José Renán Esquivel, Ciudad de Panama 0801, Panama; rchanis@hn.sld.pa; 8 Hospital Internacional La Católica, San José, Costa Rica; celina.guzman.cr@findersgroup.org; 9 Hospital Ángeles Puebla, Universidad UPAEP, Puebla 72190, Mexico; fatimaazereth.reynoso@upaep.mx; 10 Hospital de Niños Benjamín Bloom, San Salvador, El Salvador; roberto.zablah@salud.gob.sv

**Keywords:** autism spectrum disorder, disorders of gut–brain interaction, functional constipation, prevalence, Latin America, Rome IV, schoolchildren, adolescents

## Abstract

Background: Disorders of Gut–Brain Interaction (DGBIs) are present in 23.0% of the paediatric population, according to Rome IV. Latin American (LA) prevalence of DGBIs in children with Autism Spectrum Disorder (ASD) is unknown. The aim of this study was to determine the prevalence of DGBIs and possible associations in schoolchildren and adolescents with ASD from LA. Methods: An observational analytical study was conducted in LA cities. Caregivers of children with ASD completed the Rome IV Questionnaire for Pediatric Gastrointestinal Symptoms to identify DGBIs. Sociodemographic, clinical, and family variables were included. Statistical analysis involved central tendency measures, univariate and bivariate analysis, calculation odds ratios (ORs), and 95% confidence intervals (95%CIs), with *p* < 0.05 significance. Results: The study included 353 children with ASD. Predominantly male (78.8%), white (56.1%), attending private schools (79.3%), altered nutritional status (43.9%), born by c-section (57.5%), firstborn (54.7%), level of autism not classified at the time of the study (49.0%). A total of 58.9% presented DGBI. Functional constipation (FC) was the most frequent (27.2%). Those from Central America (CA) had a higher likelihood of presenting a DGBI (OR = 1.98, 95% CI = 1.25–3.12, *p* = 0.0018). Conclusions: Over half of LA schoolchildren and adolescents with ASD presented DGBI, FC being the most common, and higher likelihood of DGBI in CA.

## 1. Introduction

Disorders of Gut–Brain Interaction (DGBIs) are a group of functional gastrointestinal conditions that affect the interaction between the immune, endocrine, and nervous systems, causing discomfort and potentially localising at various levels of the digestive system [1]. The global prevalence of DGBIs in schoolchildren and adolescents is approximately 23.0% [2]. The diagnosis of these disorders, according to the Rome IV Criteria, is made through questionnaires focusing on the patient’s symptoms [3]. On the other hand, Autism Spectrum Disorder (ASD), whose multifactorial aetiology is still not fully understood, is characterised by atypical neurological development affecting communication skills, social interaction, learning, and behaviours, which tend to be repetitive, restrictive, and selective, the last two being a barrier in terms of their food selectivity and the problems that their diet will bring [4]. The treatment for these patients should be early, interdisciplinary, individualised, and adapted to the specific needs of each child and their family [5].

DGBIs have been studied in children with ASD due to the high prevalence of gastrointestinal symptoms reported in this population, which is approximately 33.0% [6]. There are several hypotheses regarding the cause of autism and its association with gastrointestinal manifestations; one of the most studied aspects of these are the DGBIs and their correlation with an imbalance in the gut microbiota, which could affect the release of proinflammatory cytokines and serotonin, factors that may help explain the behavioural manifestations observed in children diagnosed with ASD [7].

Despite the high medical comorbidity in children with ASD, there are no studies in Latin American schoolchildren and adolescents demonstrating the presence of DGBIs in these groups. This lack of knowledge represents a challenge for the accurate diagnosis and treatment of the paediatric population with ASD in the region. Therefore, the aim of this study was to determine the prevalence and possible associations of DGBIs in Central and South American schoolchildren and adolescents between 4 and 18 years old diagnosed with ASD.

## 2. Materials and Methods

An observational analytical study was conducted in which caregivers of schoolchildren and adolescents aged 4 to 18 years old diagnosed with ASD were invited to participate from capital cities in South America (SA) and Central America (CA), including Buenos Aires (Argentina), Cali (Colombia), San Jose (Costa Rica), San Salvador (El Salvador), Puebla (Mexico), and Panama City (Panama). All participants were diagnosed with ASD according to the Diagnostic and Statistical Manual of Mental Disorders (DSM-5); however, not all the children were stratified according to the level of autism at the moment of the study [5]. This study was carried out by the Functional International Digestive Epidemiological Research Survey (FINDERS) group, consisting of paediatric gastroenterologists from the Working Group DGBI of the Latin American Society of Paediatric Gastroenterology, Hepatology, and Nutrition (LASPGHAN). The focus of the study was on DGBIs in schoolchildren and adolescents with ASD. To enhance the external validity of the results, a diverse racial, ethnic, socioeconomic, and regional approach was used. This allowed for a more equitable representation of the populations from these six countries, which together account for approximately 37.0% of the total population of Latin America.

To ensure consistency and enable comparisons across multinational cross-cultural studies, the research was systematically conducted following the same methods used in previous studies by the international FINDERS consortium [8]. Additionally, the Rome Criteria were used for interviews to determine DGBIs in schoolchildren and adolescents with a diagnosis of ASD through the Questionnaire for Pediatric Gastrointestinal Symptoms Rome IV (QPGS IV), which had previously been validated by the FINDERS group in Latin American schoolchildren and adolescents [8,9]. Children with ASD included in the study were referred from paediatric gastroenterology or neurology clinics or from foundations for children with ASD in each participating country. Caregivers of schoolchildren and adolescents aged 4 to 18 years with a diagnosis of ASD were interviewed once they authorised participation in the study. The variables considered included sociodemographic (age, sex, race, origin, school), clinical (caesarean birth, prematurity, autism severity, nutritional status, comorbidities), and family-related (only child, firstborn, separated or divorced parents, intra-family DGBIs, family history of autism) factors.

Altered nutritional status was defined as all children having a Body Mass Index (BMI) outside the normal range and/or a Height-for-Age classification outside the normal parameters according to the World Health Organization, and/or a Waist Circumference above the 90th percentile, which is considered indicative of abdominal obesity by the International Diabetes Federation [10,11].

Regarding ethical considerations of each country, prior to providing a description of the study, its purpose, and the voluntary nature of participation, the parents or caregivers of the schoolchildren and adolescents signed an informed consent form in which they declared their voluntary participation and adherence to the commitments previously outlined. The study was approved by the ethics committees of Argentina (Facultad de Medicina, Universidad de Buenos Aires, 12 July 2021), Colombia (Acta 007-2021, Hospital Universitario del Valle “Evaristo García”, 12 March 2021), and El Salvador (CNEIS/2022/19, 20 September 2022).

Data collection was carried out through interviews conducted between 1 March 2021 and 28 February 2023. The data were entered into an electronic database using Excel, and to ensure accuracy in the transfer, 10% of the completed questionnaires were reviewed. The statistical analysis included information on the prevalence in each of the participating countries for initial analysis, along with central tendency measures. Additionally, the data were analysed using the two-tailed Student’s *t* test, chi-square test, and Fisher’s exact test where applicable (Stata 16 version software, College Station, TX, USA). To assess possible associations for DGBIs, univariate and bivariate analyses were performed, with the calculation of odds ratios (ORs) and their respective 95% confidence intervals (95% CIs) for each exposure variable of interest (sociodemographic, clinical, and family-related variables) and the outcome variable (presence or absence of DGBIs). A *p*-value < 0.05 was considered statistically significant. The present study assesses the internal consistency and validation of the QPGS IV in caregivers of schoolchildren and adolescents diagnosed with ASD using Cronbach’s alpha (interpretation of Cronbach’s alpha values: very high = 0.91–1.00, high = 0.61–0.80, moderate = 0.41–0.60, low = 0.21–0.40, and very low = 0.01–0.20) [12,13].

## 3. Results

Four hundred and twenty-one parents or, in some cases, caregivers of schoolchildren and adolescents aged 4 to 18 years (mean age 9.0 ± 3.7 years old) with a diagnosis of ASD were invited to participate in this study. Participants were recruited from various Latin American medical centres, foundations, and hospitals during outpatient appointments. It should be noted that all parents and caregivers had previously consented to participate in the study.

**Figure 1:**
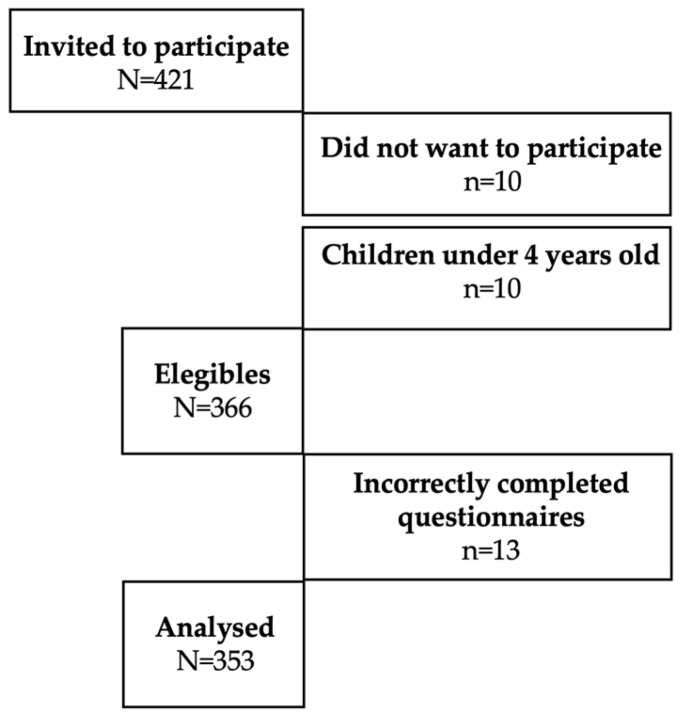
Population flowchart of the study.

Ten parents or caregivers did not want to participate. Data from respondents with inconsistent answers (n = 20) and children under 4 years of age (n = 10) were excluded. After the exclusions, N = 353 participants were analysed, of which 290 (82.2%) were schoolchildren (aged 4 to 12 years old) and 63 (17.8%) were adolescents (aged 13 to 18 years old). The population was divided into two age groups: 4–10 years old (n = 242, 68.6%) and 11–13 years old (n = 111, 31.4%) (Figure 1).

Regarding sociodemographic variables, it was found that 78.8% of the participants were male, with a male-to-female ratio of 1:3.7. The majority of participants were of white ethnicity (56.1%), followed by mixed race (40.8%). In terms of education, 79.3% of the children attended private educational institutions. Geographically, CA contributed 58.1% of the total population, mainly from Panama (15.9%), while SA contributed 41.9%, primarily from Argentina (23.2%) (Table 1).

Among the clinical variables, 57.5% of the participants were born by caesarean section, 17.3% were preterm, and 49.0% had not yet been classified by level of autism or did not know at the time of the survey. Regarding nutritional status, 43.9% of the population had an altered nutritional status. Additionally, 24.1% of the children had some comorbidity. Finally, in the family environment, 32.6% were only children, 54.7% were firstborn, and 26.1% had separated or divorced parents. A small percentage of children, 4.0%, had a family history of any DGBI, while 5.1% had a family history of autism (Table 2).

### 3.1. Prevalence

Among the 353 schoolchildren and adolescents with ASD assessed using the Rome IV Criteria, more than half (58.9%) met the criteria for at least one DGBI. The most frequent DGBI was functional constipation (FC) (27.2%), followed by functional dyspepsia (FD) (21.8%), unspecified abdominal pain (4.5%), and disorders associated with nausea and vomiting (2.9%) (Table 3).

### 3.2. Possible Associations

A possible association was found between the factors assessed and geographical location. Specifically, schoolchildren and adolescents with ASD from CA were more likely to have some DGBIs (OR = 1.98, 95% CI= 1.25–3.12, *p* = 0.0018) compared to those from SA. Within CA, Mexico also showed a significant association (OR = 1.95, 95% CI = 0.98–4.09, *p* = 0.0425). However, no significant differences were observed when analysing other sociodemographic, clinical, and family variables (*p* > 0.05) (Table 4).

### 3.3. Validation and Internal Consistency of the QPGS IV in Spanish for ASD

Validation and internal consistency analysis of the questionnaire, designed for parents and caregivers of schoolchildren and adolescents with ASD, showed a Cronbach’s alpha of 0.7818 (high). When the QPGS IV by section was analysed, interpretations ranged from moderate to high (Table 5).

### 3.4. Main Digestive Symptoms Identified

When evaluating the main digestive symptoms according to the Rome IV questionnaire, it was found that most children presented flatus (50.4%), followed by painful stools (35.7%), large stools (30.6%), history of large faecal mass in rectum (26.6%), and hard stools (23.8%) (Table 6).

## 4. Discussion

In the present study, we aimed to determine the prevalence of DGBIs in adolescents with ASD from CA and SA. Due to the inherent limitations in administering the questionnaires to the target population, it was the parents or guardians who completed the QPGS IV, which demonstrated high validity and reliability (Cronbach’s alpha of 0.7818) (Table 5). This questionnaire is considered the gold standard for diagnosing DGBIs in children and adults, despite being viewed as subjective [3], has adequate construct validity [9], a sensitivity of 75% (95% CI = 59.0–79.0), and a specificity of 90% (95% CI = 83.0–98.0) [8], and it is recommended that it be guided to improve internal reliability [14].

We found that Latin American schoolchildren and adolescents diagnosed with ASD had a prevalence of 58.9% for presenting some type of DGBI according to the Rome IV Criteria. This figure is higher than those reported in similar age groups of children without ASD: A systematic review of European, North American and Latin American children between the ages of 4 and 18, analysed according to Rome IV Criteria, found that the global prevalence of DGBI was 23.0% (95% CI = 21.0–25.0%, I^2^ = 99.0%) [2], in a cross-sectional study made in Colombia, the prevalence of these disorders was 21.2% [15], 22.3% in Ecuador [15], 25.0% in the United States [16], 31.2% in Bosnia and Herzegovina [17], and between 26.2 and 26.4% in Italy [18]. Some authors compared the prevalence of these disorders according to the Rome III Criteria, and the prevalence ranged from 13.4 to 29.9% (higher compared to Rome IV prevalence) in Latin American countries such as Mexico, Colombia, El Salvador, Ecuador, and Panama [15].

Our prevalence of DGBIs is lower than that reported by Gülpınar et al. [19], who, in a case–control study conducted with Turkish children aged 4 to 10 years old, found a prevalence of 76.5% in children with ASD using the Rome III questionnaire. Even in a subanalysis to compare with our Latin American children of the same age (76.5% versus 59.1%, *p* = 0.006), the difference is notable and could be due to the questionnaire they applied or to cultural factors that may influence the prevalence of these DGBIs, such as eating habits.

In the present study and according to Rome IV, the most prevalent DGBI in Latin American schoolchildren and adolescents with ASD was FC, which occurred in 27.2%. This was higher than reported in neurotypical children of the same age according to Rome IV in European countries, Latin America, and the United States of America [2,15,16,17,18], and according to Rome III for Latin American countries [15], but lower than the figures found in Bosnia and Herzegovina [17] according to Rome IV and those identified by Gülpınar et al. [19], who found 38.2% in Turkish children with ASD.

Our second most prevalent DGBI was FD with 21.9%, higher than those reported according to Rome IV in healthy children without ASD [2,15,16,17,18] and with ASD [19], as well as according to Rome III Criteria in healthy Latin American children without ASD [2,15,16,17,18]. These differences could be explained by the hypothesis of a bidirectional interaction between the central nervous system and the digestive tract [20], as well as methodologically, by the type of questionnaire and its interpretation, the coexistence of DGBIs, the sample size or regional, cultural, nutritional, and lifestyle differences, among others.

In future research, it would be beneficial to investigate the potential causes of the high prevalence of FC in children with ASD. These causes may include, but are not limited to, the child’s diet and lifestyle. Furthermore, it may be advantageous to propose new non-invasive and cost-effective therapies that have demonstrated encouraging results in children with FC, such as transcutaneous tibial nerve stimulation [21,22] or novel drugs such as Linaclotide [23,24,25].

Regarding drug treatment for the most prevalent DGBI, a three-phase study proved that Linaclotide is safe and effective in treating FC [23,24,25]. It evaluated multiple doses and responses in patients aged between 6 and 17 years old. It also evaluated the dose and response in patients aged between 2 and 5 years old [23,24,25] and concluded with a double-blind, placebo-controlled, multicentre trial in children aged 6 to 17 years [23,24,25]. Subsequent to these findings, the drug has been approved by the Food and Drugs Administration in the United States of America as a treatment for children suffering from FC, and the only adverse effect found was moderate to mild diarrhoea in some of the patients [23,24,25].

The only possible association that we found in this group of Latin American schoolchildren and adolescents with ASD was the relevance of the geographical region they came from, as there was a higher prevalence of DGBIs in CA (OR = 1.98, 95% CI = 1.25–3.12, *p* = 0.0018), specifically in Mexico, and a lower prevalence in SA, particularly in Colombia. Several hypotheses could explain this, including nutritional psycho-affective factors, the role of the microbiota, and access to health care.

From a nutritional standpoint, we did not find differences in their nutritional status, even in the most prevalent DGBI, FC. However, it is worth noting that nearly one-third of the children with both FC and ASD (26.0%) had altered nutritional status. Regarding what we previously mentioned, there is a theory that neurotypical children, no matter their sex, who are overweight or obese may experience FC, and the risk of obesity is higher in children with FC; those risks were observed especially in developed countries (95% CI = 1.49–3.46; *p* = 0.000), but not in developing countries [26]; however, these results have not been confirmed in Colombian children without ASD with certain DGBI, such as FC [27]. Although the present study did not collect data on specific dietary patterns followed by each patient, recent studies have shown that certain food components such as fermentable oligosaccharides, disaccharides, monosaccharides, and polyol (FODMAPs), as well as gluten, have been associated with exacerbation of gastrointestinal symptoms in the neurotypical population [28]. Diets high in FODMAPs and/or gluten may negatively impact gastrointestinal function, leading to symptoms including diarrhoea, abdominal bloating, and pain [28,29]. Evidence suggests that such dietary interventions may be beneficial, particularly in children presenting with abdominal pain and/or constipation [29]. Furthermore, other studies indicate that gluten and casein-free diets may also contribute to symptom improvement in patients with ASD [30]. Due to knowledge gaps, future research must include detailed diet data from children with ASD from each country, knowing that avoidant/restrictive food intake disorder is common in this population and has a negative impact on multiple aspects of their lives [31].

Despite the absence of any possible psycho-affective associations in these Latin American schoolchildren and adolescents with ASD and DGBI, there was a higher percentage of divorced or separated parents in Latin American adolescents with ASD compared to schoolchildren (38.7% vs. 20.2%, *p* = 0.000). This finding may be indicative of the psychosocial and familial strain involved in raising children with ASD in a society that is poorly adapted to the needs of this population. In correlation with our findings, one study showed that parents of children with ASD had lower scores than parents of neurotypical children on the WHOQOL-BREF quality of life scale and the QOL [32,33]. In future research, it would be beneficial to design and conduct a study that assesses family quality of life in the Latin American population. This study should be conducted before and after the diagnosis of the child with ASD, as well as following the diagnosis and interventions for both the family and the child.

Research related to alterations in the microbiota and behaviour in patients with ASD commenced reporting in 2015 [34], following the case report of a young individual from the United States of America who exhibited behavioural improvements regarding social interaction, such as eye contact, speech improvement, and motricity, after undergoing common antibiotic treatment [34]. Subsequent studies have demonstrated significant disparities in the microbiota of children with ASD in comparison to those with typical development [35,36]. The results of most studies have documented a low presence of beneficial bacteria and a higher abundance of harmful bacteria, such as *Clostridium,* in patients with ASD compared to neurotypical children. The presence of these bacteria has been related to gastrointestinal problems in children with ASD [37,38,39,40]. However, these findings regarding the microbiota of children with ASD should not be generalised because the microbiota varies from patient to patient according to diet, lifestyle, and eating habits [41,42]. These findings have led to new research assessing the prospective therapeutic benefits of pre- and probiotics [43,44], as well as faecal transplants [45,46], meticulously tailored to the individual requirements of each patient [35].

Another potential cofactor in this group of patients and their families is access to health care. Although there are no studies conducted in Latin America, a recent systematic review highlights disparities related to health care settings, screening and referral process, diagnosis, services, navigating the health system, insurance, prescription, language and cultural barriers, low quality of health interactions, stereotypes and discrimination, stigma from family and the community, and the intersection of multiple barriers when accessing health care services [47].

In these Latin American schoolchildren and adolescents with ASD, between 4.5% and 50.4% presented one or more symptoms based on the Rome IV Pediatric Digestive Symptom Questionnaire (Table 6). Children with ASD are 2 [48] to 4 times [49] more likely to present digestive symptoms, as well as multiple digestive symptoms (30.6% versus 5.4%, *p* < 0.05) [50], compared to children without ASD, which impacts both their quality of life and that of their families [51]. As a result, non-verbal scales have been proposed to identify digestive symptoms and assess their psychometric characteristics for both research and clinical purposes [52]. Many of the digestive symptoms in patients with ASD are linked to their stereotyped behaviours, with a marked food selectivity and a strong preference for carbohydrates and specific foods [53]. In a case–control study comparing typically developing children and children with ASD, gastrointestinal symptoms were associated, in both groups, with an increase in self-injurious behaviour, restricted stereotyped behaviour, aggressive behaviour, sleep problems, attention problems, somatic complaints and an increase in parasomnias [50]. Children with ASD and gastrointestinal symptoms were also more likely to experience the problems listed above than children with ASD but without gastrointestinal symptoms [50].

We consider the strength of our study to be the diversification of the population, which represents approximately 37.0% of the total population of Latin America. Upon calculating Cronbach’s alpha for the questionnaire completed by the parents or caregivers of children with ASD, we concluded that this method of administering the questionnaire to third parties was valid and reliable (Cronbach’s alpha = 0.7818). One of the limitations of our study was that most of the children included were from private schools, which could introduce a socioeconomic bias. However, to mitigate this bias, a statistical adjustment was performed by stratifying according to the type of educational institution. We acknowledge that one area for improvement is the need to expand the sample size in certain countries. Additionally, we cannot rule out that the results obtained may not be fully generalisable to all of Latin America. Furthermore, it is necessary to investigate the dietary habits of each child in order to analyse the effects related to DGBIs and gut microbiota. As this is the first Latin American study to evaluate the prevalence of DGBIs in children with ASD, the lack of previous similar research makes it difficult to compare our findings. We propose, in the future, to conduct a case–control study at the Latin American level to more accurately identify the risk factors affecting the school-aged and adolescent population with ASD.

## 5. Conclusions

In conclusion, through this study and in comparison with the literature reviews of studies conducted on Latin American children and those from other continents, the hypothesis can be confirmed that there is a higher prevalence of DGBIs in children with ASD compared to those with neurotypical development. In this group of Latin American schoolchildren and adolescents with ASD, regardless of their level of autism, 6 out of every 10 children present some form of DGBI, with FC being the most frequent. Additionally, children from CA have a higher likelihood of presenting any DGBI.

## Figures and Tables

**Table 1:** Demographics.

	All (N = 353)	4–10 Years Old (n = 242)	11–18 Years Old (n = 111)
**Sociodemographic variables**			
**Age (years)**			
Mean SD	9.0 ± 3.7	6.8 ± 1.8	13.7 ± 2.4
Range	4–18	4–10	11–18
**Age groups (years) n (%)**			
Schoolchildren (8–12)	290 (82.2)	242 (100.0)	48 (43.2)
Adolescents (13–18)	63 (17.8)	0 (0.0)	63 (56.8)
**Sex n (%)**			
Female	75 (21.3)	47 (19.4)	28 (25.2)
Male	278 (78.8)	195 (80.6)	83 (74.8)
**Race n (%)**			
White	198 (56.1)	132 (54.5)	66 (59.5)
Mixed race	144 (40.8)	100 (41.3)	44 (39.6)
Afro-descendant	7 (2.0)	7 (2.9)	0 (0.0)
Indigenous	4 (1.1)	3 (1.2)	1 (0.9)
**Country n (%)**			
**South America**	148 (41.9)	102 (42.1)	46 (41.4)
Argentina	82 (23.2)	61 (25.2)	21 (18.9)
Colombia	66 (18.7)	41 (16.9)	25 (22.5)
**Central America**	205 (58.1)	140 (57.9)	65 (58.6)
Costa Rica	52 (14.7)	33 (13.6)	19 (17.1)
El Salvador	47 (13.3)	39 (16.1)	8 (7.2)
Mexico	50 (14.2)	30 (12.4)	20 (18.0)
Panama	56 (15.9)	38 (15.7)	18 (16.2)
**Type of school n (%)**			
Public	48 (13.6)	31 (12.8)	17 (15.3)
Private	280 (79.3)	192 (79.3)	88 (79.3)
Does not attend school	25 (7.1)	19 (7.9)	6 (5.4)

SD = Standard Deviation.

**Table 2:** Clinical and family variables.

	All (N = 353)	4–10 Years Old (n = 242)	11–18 Years Old (n = 111)	*p*
**Clinical variables n (%)**				
**Caesarean section**				
No	150 (42.5)	98 (40.5)	52 (46.9)	0.158
Yes	203 (57.5)	144 (59.5)	59 (53.1)	
**Prematurity**				
No	292 (82.7)	202 (83.5)	90 (81.1)	0.341
Yes	61 (17.3)	40 (16.5)	21 (18.9)	
**Level of autism according to DSM-5**				
**I**				
No	99 (28.1)	67 (27.7)	32 (28.8)	0.552
Yes	81 (22.9)	55 (22.7)	26 (23.4)	
**II**				
No	110 (31.2)	74 (30.6)	36 (32.4)	0.494
Yes	70 (19.8)	48 (19.8)	22 (19.8)	
**III**				
No	151 (42.8)	103 (42.6)	48 (43.2)	0.466
Yes	29 (8.2)	19 (7.9)	10 (9.0)	
**Not classified yet or do not know at the time of the study**				
No	180 (51.0)	122 (50.4)	58 (52.2)	0.418
Yes	173 (49.0)	120 (49.6)	53 (47.8)	
**Comorbidity**				
No	268 (75.9)	188 (77.7)	80 (72.1)	0.156
Yes	85 (24.1)	54 (22.3)	31 (27.9)	
**Altered nutritional status**				
No	198 (56.1)	134 (55.4)	64 (57.7)	0.388
Yes	155 (43.9)	108 (44.6)	38 (42.3)	
**Family variables n (%)**				
**Only child**				
No	238 (67.4)	158 (65.3)	80 (72.1)	0.127
Yes	115 (32.6)	84 (34.7)	31 (27.9)	
**Firstborn**				
No	160 (45.3)	113 (46.7)	47 (42.3)	0.259
Yes	193 (54.7)	129 (53.3)	64 (57.7)	
**Separated/divorced parents**				
No	261 (73.9)	193 (79.8)	68 (61.3)	0.000
Yes	92 (26.1)	49 (20.2)	43 (38.7)	
**DGBIs intra-family**				
No	339 (96.0)	232 (95.9)	107 (96.4)	0.536
Yes	14 (4.0)	10 (4.1)	4 (3.6)	
**Autism in the family**				
No	335 (94.9)	230 (95.0)	105 (94.6)	0.521
Yes	18 (5.1)	12 (5.0)	6 (5.4)	

DGBIs = Disorders of Gut–Brain Interaction; DSM-5 = Diagnostic and Statistical Manual of Mental Disorders Fifth Edition.

**Table 3:** Prevalence of DGBIs.

	All (N = 353)	4–10 Years Old (n = 242)	11–18 Years Old (n = 111)
**DGBIs n (%)**			
No	145 (41.1)	99 (40.9)	46 (41.4)
Yes	208 (58.9)	143 (59.1)	65 (58.6)
**Associated with nausea and vomiting**	**10 (2.9)**	**6 (2.4)**	**4 (3.6)**
Functional vomiting	6 (1.7)	2 (0.8)	4 (3.6)
Cyclic vomiting	1 (0.3)	1 (0.4)	0 (0.0)
Rumination	2 (0.6)	2 (0.8)	0 (0.0)
Aerophagia	1 (0.3)	1 (0.4)	0 (0.0)
**Associated with abdominal pain**	**100 (28.3)**	**69 (28.5)**	**31 (27.9)**
Functional dyspepsia	77 (21.8)	55 (22.7)	22 (19.8)
Postprandial	74 (21.0)	53 (21.9)	21 (18.9)
Epigastric	2 (0.6)	1 (0.4)	1 (0.9)
Irritable bowel syndrome	5 (1.4)	5 (2.1)	0 (0.0)
IBS with diarrhoea	1 (0.3)	1 (0.4)	0 (0.0)
IBS with constipation	4 (1.1)	4 (1.7)	0 (0.0)
Abdominal migraine	2 (0.6)	1 (0.4)	1 (0.9)
Functional abdominal pain not otherwise specified	16 (4.5)	8 (3.3)	8 (7.2)
**Associated with defecation**	**98 (27.8)**	**68 (28.1)**	**30 (27.0)**
Functional constipation	96 (27.2)	67 (27.7)	29 (26.1)
Non-retentive faecal incontinence	2 (0.6)	1 (0.4)	1 (0.9)

DGBIs = Disorders of Gut–Brain Interaction; IBS = irritable bowel syndrome.

**Table 4:** Possible associations with DGBIs.

	DGBIs		OR	95% CI	*p*
	No	Yes			
	145 (41.1)	208 (58.9)			
Sociodemographic variables					
**Age groups**					
Schoolchildren	120 (82.8)	170 (81.7)	1.00		0.8040
Adolescents	25 (17.2)	38 (18.3)	1.07	0.59–1.95	
**Sex**					
Female	31 (21.4)	44 (21.2)	1.00		0.9594
Male	114 (78.6)	164 (78.8)	1.01	0.58–1.75	
**Race**					
**White**					
No	64 (44.1)	91 (43.8)	1.00		0.9424
Yes	81 (55.9)	117 (56.2)	1.01	0.64–1.59	
**Mixed race**					
No	91 (62.8)	118 (56.7)	1.00		0.2569
Yes	54 (37.2)	90 (43.3)	1.28	0.81–2.03	
**Afro-descendant**					
No	138 (95.2)	208 (100.0)	n/a		
Yes	7 (4.8)	0 (0.0)			
**Indigenous**					
No	142 (97.9)	207 (99.5)	1.00		0.1655
Yes	3 (2.1)	1 (0.5)	0.22	0.004–2.89	
**Type of school**					
**Public**					
No	122 (84.1)	183 (88.0)	1.00		0.3001
Yes	23 (15.9)	25 (12.0)	0.72	0.37–1.40	
**Private**					
No	34 (23.5)	39 (18.7)	1.00		0.2836
Yes	111 (76.5)	169 (81.3)	1.32	0.76–2.30	
**Does not attend school**					
No	134 (94.4)	194 (93.3)	1.00		0.7579
Yes	11 (5.6)	14 (6.7)	0.87	0.35–2.21	
**Country**					
**South America**					
No	70 (48.3)	135 (64.9)	1.00		0.0018
Yes	75 (51.7)	73 (35.1)	0.50	0.31–0.79	
**Argentina**					
No	110 (75.9)	161 (77.4)	1.00		0.7358
Yes	35 (24.1)	47 (22.6)	0.91	0.54–1.56	
**Colombia**					
No	105 (72.4)	182 (87.5)	1.00		0.0003
Yes	40 (27.6)	26 (12.5)	0.37	0.20–0.67	
**Central America**					
No	75 (51.7)	73 (35.1)	1.00		0.0018
Yes	70 (48.3)	135 (64.9)	1.98	1.25–3.12	
**Panama**					
No	126 (86.9)	171 (82.2)	1.00		0.2359
Yes	19 (13.1)	37 (17.8)	1.43	0.76–2.77	
**El Salvador**					
No	123 (84.3)	183 (88.0)	1.00		0.3909
Yes	22 (15.2)	25 (12.0)	0.76	0.39–1.49	
**Costa Rica**					
No	130 (89.7)	171 (82.2)	1.00		0.0522
Yes	15 (10.3)	37 (17.8)	1.87	0.95–3.83	
**Mexico**					
No	131 (90.3)	172 (82.7)	1.00		0.0425
Yes	14 (9.7)	36 (17.3)	1.95	0.98–4.09	
**Clinical variables**					
**Caesarean section**					
No	58 (40.0)	92 (44.2)	1.00		0.4289
Yes	87 (60.0)	116 (55.8)	0.84	0.53–1.32	
**Prematurity**					
No	118 (81.4)	174 (83.7)	1.00		0.5782
Yes	27 (18.6)	34 (16.4)	0.85	0.47–1.55	
**Level of autism according to DSM-5**					
**I**					
No	37 (56.9)	62 (53.9)	1.00		0.6966
Yes	28 (43.1)	53 (46.1)	1.12	0.58–2.18	
**II**					
No	39 (60.0)	71 (61.7)	1.00		0.8182
Yes	26 (40.0)	44 (38.3)	0.92	0.47–1.82	
**III**					
No	54 (83.1)	97 (84.4)	1.00		0.8237
Yes	11 (16.9)	18 (15.6)	0.91	0.37–2.30	
**Not classified yet or do not know at the time of the study**					
No	65 (44.8)	115 (55.3)	1.00		0.0531
Yes	80 (55.2)	93 (44.7)	0.65	0.41–1.02	
**Comorbidity**					
No	112 (77.2)	156 (75.0)	1.00		0.6280
Yes	33 (22.8)	52 (25.0)	1.13	0.66–1.93	
**Altered nutritional status**					
No	84 (57.9)	114 (54.8)	1.00		0.5607
Yes	61 (42.1)	94 (45.2)	1.13	0.72–1.78	
**Family variables**					
**Only child**					
No	97 (66.9)	141 (67.8)	1.00		0.8604
Yes	48 (33.1)	67 (32.2)	0.96	0.59–1.55	
**Firstborn**					
No	67 (46.2)	93 (44.7)	1.00		0.7813
Yes	78 (53.8)	115 (55.3)	1.06	0.67–1.66	
**Separated/divorced parents**					
No	110 (75.9)	151 (72.6)	1.00		0.4916
Yes	35 (24.1)	57 (27.4)	1.18	0.70–1.99	
**DGBIs intra-family**					
No	142 (97.9)	197 (94.7)	1.00		0.1273
Yes	3 (2.1)	11 (5.3)	2.64	0.67–14.98	
**Autism in the family**					
No	138 (95.2)	197 (94.7)	1.00		0.8464
Yes	7 (4.8)	11 (5.3)	1.10	0.37–3.43	

DGBIs = Disorders of Gut–Brain Interaction; DSM-5 = Diagnostic and Statistical Manual of Mental Disorders Fifth Edition.

**Table 5:** Validation and internal consistency of the QPGS IV in Spanish designed for parents and caregivers of schoolchildren and adolescents with ASD.

	Alpha De Cronbach	Interpretation
**Total questionnaire**	0.7818	High
**Section A**	0.7331	High
**Section B**	0.4706	Moderate
**Section C**	0.6535	High
**Section D**	0.6110	High
**Section E**	0.6367	High

QPGS IV = Questionnaire for Pediatric Gastrointestinal Symptoms Rome IV.

**Table 6:** Main digestive symptoms identified using QPGS IV.

Symptom	n (%)
Flatus	178 (50.4)
Painful stool	126 (35.7)
Large stools	108 (30.6)
History of large faecal mass in rectum	94 (26.6)
Hard stools	84 (23.8)
Belching	84 (23.8)
Stool retention	78 (22.1)
Soiling	73 (20.7)
Abdominal pain around and below belly button	68 (19.3)
Early satiation	66 (18.7)
Abdominal pain above belly button	53 (15.0)
Abdominal distension	48 (13.6)
Watery stools	28 (7.9)
Swallowing air	26 (7.4)
Nausea	25 (7.1)
Heartburn	16 (4.5)

QPGS IV = Questionnaire for Pediatric Gastrointestinal Symptoms Rome IV.

## Data Availability

The data presented in this study are available on request from the corresponding author. The data are not publicly available due to privacy and ethical restrictions.

## Abbreviations

The following abbreviations are used in this manuscript:

DGBIs	Disorders of Gut–Brain Interaction
ASD	Autism Spectrum Disorder
SA	South America
CA	Central America
OR	Odds ratio
BMI	Body Mass Index
FC	Functional constipation
QPGS IV	Questionnaire for Pediatric Gastrointestinal Symptoms Rome IV
FINDERS	Functional International Digestive Epidemiological Research Survey
DSM-V	Diagnostic Statistical Manual of Mental Disorders
LASPGHAN	Latin America Society of Paediatric Gastroenterology, Hepatology, and Nutrition
FD	Functional dyspepsia
FAP	Functional abdominal pain

## References

[1] Kasarello K., Cudnoch-Jedrzejewska A., Czarzasta K. (2023). Communication of gut microbiota and brain via immune and neuroendocrine signaling. Front. Microbiol..

[2] Velasco-Benítez C.A., Collazos-Saa L.I., García-Perdomo H.A. (2022). A systematic review and meta-analysis in schoolchildren and adolescents with functional gastrointestinal disorders according to Rome IV criteria. Arq. Gastroenterol..

[3] Drossman D.A. (2016). Functional gastrointestinal disorders: History, pathophysiology, clinical features and Rome IV. Gastroenterology.

[4] Dargenio V.N., Dargenio C., Castellaneta S., De Giacomo A., Laguardia M., Schettini F., Francavilla R., Cristofori F. (2023). Intestinal barrier dysfunction and microbiota-gut-brain axis: Possible implications in the pathogenesis and treatment of autism spectrum disorder. Nutrients.

[5] American Psychiatric Association (2014). Diagnostic and Statistical Manual of Mental Disorders.

[6] Lasheras I., Real-López M., Santabárbara J. (2023). Prevalence of gastrointestinal symptoms in autism spectrum disorder: A meta-analysis. An. Pediatr..

[7] Morton J.T., Jin D.-M., Mills R.H., Shao Y., Rahman G., McDonald D., Zhu Q., Balaban M., Jiang Y., Cantrell K. (2023). Multi-level analysis of the gut-brain axis shows autism spectrum disorder-associated molecular and microbial profiles. Nat. Neurosci..

[8] Velasco-Benítez C.A., Ortíz-Rivera C.J., Sánchez-Pérez M.P., Játiva-Mariño E., Villamarín-Betancourt E.A., Saps M. (2019). Utilidad de los cuestionarios de Roma IV en español para identificar desórdenes gastrointestinales funcionales en pediatría. Grupo de trabajo de la Sociedad Latinoamericana de Gastroenterología, Hepatología y Nutrición Pediátrica (SLAGHNP). Acta Gastroenterol. Latinoam..

[9] Saps M., Nichols-Vinueza D.X., Mintjens S., Pusatcioglu C.K., Velasco-Benítez C.A. (2014). Construct validity of the pediatric Rome III criteria. J. Pediatr. Gastroenterol. Nutr..

[10] Zimmet P., Alberti K.G.M., Kaufman F., Tajima N., Silink M., Arslanian S., Wong G., Bennet P., Shaw J., Caprio S. (2007). The metabolic syndrome in children and adolescents—An IDF consensus report. Pediatr. Diabetes.

[11] Vargas M.E., Souki A., Ruiz G., García D., Mengual E., Gonzalez C.C., Chavez M., Gonzalez M. (2011). Percentiles de circunferencia de cintura en niños y adolescentes del municipio de Maracaibo del Estado Zulia, Venezuela. An. Venez..

[12] Collins L.M. (2007). Research Design and Methods.

[13] Barbosa E.Y. (2021). A neurodidactic model for teaching elementary EFL students in a college context. Engl. Lang. Teach..

[14] Baaleman D.F., Velasco-Benítez C.A., Méndez-Guzmán L.M., Benninga M.A., Saps M. (2021). Can we rely on the Rome IV questionnaire to diagnose children with functional gastrointestinal disorders?. J. Neurogastroenterol. Motil..

[15] Velasco-Benítez C. (2022). Trastornos Digestivos Funcionales En Pediatría.

[16] Robin S.G., Keller C., Zwiener R., Hyman P.E., Nurko S., Saps M., Di Lorenzo C., Shulman R.J., Hyams J.S., Palsson O. (2018). Prevalence of pediatric functional gastrointestinal disorders utilizing the Rome IV criteria. J. Pediatr..

[17] Selimović A., Mekić N., Terzić S., Ćosićkić A., Zulić E., Mehmedović M. (2024). Functional gastrointestinal disorders in children: A single centre experience. Med. Glas..

[18] Cenni S., Pensabene L., Dolce P., Campanozzi A., Salvatore S., Pujia R., Serra M.R., Scarpato E., Miele E., Staiano A. (2023). Prevalence of functional gastrointestinal disorders in Italian children living in different regions: Analysis of the difference and the role of diet. Dig. Liver Dis..

[19] Aydın Ö.G., Baykara H.B., Akın K., Kahveci S., Şeker G., Güler Y., Öztürk Y. (2024). Evaluation of functional gastrointestinal disorders in children aged 4–10 years with autism spectrum disorder. Turk. J. Pediatr..

[20] Saurman V., Margolis K.G., Luna R.A. (2020). Autism spectrum disorder as a brain-gut-microbiome axis disorder. Dig. Dis. Sci..

[21] Rego R.M.P., Machado N.C., Carvalho M.A., Graffunder J.S., Ortolan E.V.P., Lourenção P.L.T.A. (2019). Transcutaneous posterior tibial nerve stimulation in children and adolescents with functional constipation: A protocol for an interventional study. Medicine.

[22] Yu Z.T., Song J.M., Qiao L., Wang Y., Chen Y., Wang E.H., Zhang S.C. (2023). A randomized, double-blind, controlled trial of percutaneous tibial nerve stimulation with pelvic floor exercises in the treatment of childhood constipation. Am. J. Gastroenterol..

[23] Di Lorenzo C., Nurko S., Hyams J.S., Rodriguez-Araujo G., Almansa C., Shakhnovich V., Saps M., Simon M. (2024). Randomized controlled trial of linaclotide in children aged 6-17 years with functional constipation. J. Pediatr. Gastroenterol. Nutr..

[24] Di Lorenzo C., Robert J., Rodriguez-Araujo G., Shakhnovich V., Xie W., Nurko S., Saps M. (2024). Safety and efficacy of linaclotide in children aged 2–5 years with functional constipation: Phase 2, randomized study. J. Pediatr. Gastroenterol. Nutr..

[25] Di Lorenzo C., Khlevner J., Rodriguez-Araujo G., Xie W., Huh S.Y., Ando M., Hyams J.S., Nurko S., Benninga M.A., Simon M. (2024). Efficacy and safety of linaclotide in treating functional constipation in paediatric patients: A randomised, double-blind, placebo-controlled, multicentre, phase 3 trial. Lancet Gastroenterol. Hepatol..

[26] Wang G.N., Zhang K., Xiong Y.Y., Liu S. (2023). The relationship between functional constipation and overweight/obesity in children: A systematic review and meta-analysis. Pediatr. Res..

[27] Koppen I.J.N., Velasco-Benítez C.A., Benninga M.A., Di Lorenzo C., Saps M. (2016). Is there an association between functional constipation and excessive bodyweight in children?. J. Pediatr..

[28] Hill P., Muir J.G., Gibson P.R. (2017). Controversies and Recent Developments of the Low-FODMAP Diet. Gastroenterol. Hepatol..

[29] Hakime N., Walton J., Roberts K.M., Nahikian-Nelms M., Witwer A.N. (2021). The Effect of the Low FODMAP Diet on Gastrointestinal Symptoms, Behavioral Problems and Nutrient Intake in Children with Autism Spectrum Disorder: A Randomized Controlled Pilot Trial. J. Autism Dev. Disord..

[30] Saad K., Shabaan I., Hassan A.E.M.M., Ezzat M., Abouzed M.A., Hamed Y., Fahmy M., Gad E.F. (2024). Gluten-Free, Casein-Free Diet for Children with Autism Spectrum Disorder: A Case-Controlled Study. J. Pharm. Biallied Sci..

[31] Bourne L., Mandy W., Bryant-Waugh R. (2022). Avoidant/restrictive food intake disorder and severe food selectivity in children and young people with autism: A scoping review. Dev. Med. Child. Neurol..

[32] Raju S., Hepsibah P.E.V., Niharika M.K. (2023). Quality of life in parents of children with Autism spectrum disorder: Emphasizing challenges in the Indian context. Int. J. Dev. Disabil..

[33] Musetti A., Manari T., Dioni B., Raffin C., Bravo G., Mariani R., Esposito G., Dimitriou D., Plazzi G., Franceschini C. (2021). Parental Quality of Life and Involvement in Intervention for Children or Adolescents with Autism Spectrum Disorders: A Systematic Review. J. Pers. Med..

[34] Rodakis J. (2015). An n=1 case report of a child with autism improving on antibiotics and a father’s quest to understand what it may mean. Microb. Ecol. Health Dis..

[35] Iglesias-Vázquez L., Van Ginkel Riba G., Arija V., Canals J. (2020). Composition of gut microbiota in children with autism spectrum disorder: A systematic review and meta-analysis. Nutrients.

[36] Lin P., Zhang Q., Sun J., Li Q., Li D., Zhu M., Fu X., Zhao L., Wang M., Lou X. (2024). A comparison between children and adolescents with autism spectrum disorders and healthy controls in biomedical factors, trace elements, and microbiota biomarkers: A meta-analysis. Front. Psychiatry.

[37] Ma B., Liang J., Dai M., Wang J., Luo J., Zhang Z., Jing J. (2019). Altered Gut Microbiota in Chinese Children With Autism Spectrum Disorders. Front. Cell Infect. Microbiol..

[38] Wang L., Christophersen C.T., Sorich M.J., Gerber J.P., Angley M.T., Conlon M.A. (2011). Low relative abundances of the mucolytic bacterium Akkermansia muciniphila and Bifidobacterium spp. in feces of children with autism. Appl. Environ. Microbiol..

[39] Strati F., Cavalieri D., Albanese D., De Felice C., Donati C., Hayek J., Jousson O., Leoncini S., Renzi D., Calabrò A. (2017). New evidences on the altered gut microbiota in autism spectrum disorders. Microbiome.

[40] Argou-Cardozo I., Zeidán-Chuliá F. (2018). Clostridium Bacteria and Autism Spectrum Conditions: A Systematic Review and Hypothetical Contribution of Environmental Glyphosate Levels. Med. Sci..

[41] Zang P. (2022). Influence of Foods and Nutrition on the Gut Microbiome and Implications for Intestinal Health. Int. J. Mol. Sci..

[42] Berding K., Donovan S.M. (2018). Diet Can Impact Microbiota Composition in Children With Autism Spectrum Disorder. Front. Neurosci..

[43] Sanctuary M.R., Kain J.N., Chen S.Y., Kalanetra K., Lemay D.G., Rose D.R., Yang H.T., Tancredi D.J., German J.B., Slupsky C.M. (2019). Pilot study of probiotic/colostrum supplementation on gut function in children with autism and gastrointestinal symptoms. PLoS ONE.

[44] Patusco R., Ziegler J. (2018). Role of probiotics in managing gastrointestinal dysfunction in children with autism spectrum disorder: An update for practitioners. Adv. Nutr..

[45] Dossaji Z., Khattak A., Tun K.M., Hsu M., Batra K., Hong A.S. (2023). Efficacy of fecal microbiota transplant on behavioral and gastrointestinal symptoms in pediatric autism: A systematic review. Microorganisms.

[46] Yu Y., Wang W., Zhang F. (2023). The next generation fecal microbiota transplantation: To transplant bacteria or virome. Adv. Sci..

[47] Lindsay S., Li Y., Joneja S., Hsu S. (2025). Experiences of racism and racial disparities in health care among children and youth with autism and their caregivers: A systematic review. Disabil. Rehabil..

[48] Lai K.Y.C., Leung P.W.L., Hung S.F., Shea C.K.S., Mo F., Che K.K.I., Tse C.Y., Lau F.L.F., Ma S.L., Wu J.C.Y. (2020). Gastrointestinal problems in Chinese children with autism spectrum disorder. Neuropsychiatr. Dis. Treat..

[49] Gan H., Su Y., Zhang L., Huang G., Lai C., Lv Y., Li Y. (2023). Questionnaire-based analysis of autism spectrum disorders and gastrointestinal symptoms in children and adolescents: A systematic review and meta-analysis. Front. Pediatr..

[50] Restrepo B., Angkustsiri K., Taylor S.L., Rogers S.J., Cabral J., Heath B., Hechtman A., Solomon M., Ashwood P., Amaral D.G. (2020). Developmental-behavioral profiles in children with autism spectrum disorder and co-occurring gastrointestinal symptoms. Autism Res..

[51] Holingue C., Poku O., Pfeiffer D., Murray S., Fallin M.D. (2022). Gastrointestinal concerns in children with autism spectrum disorder: A qualitative study of family experiences. Autism.

[52] Holingue C., Kalb L.G., Musci R., Lukens C., Lee L.-C., Kaczaniuk J., Landrum M., Buie T., Fallin M.D. (2022). Characteristics of the autism spectrum disorder gastrointestinal and related behaviors inventory in children. Autism Res..

[53] Bandini L.G., Curtin C., Phillips S., Anderson S.E., Maslin M., Must A. (2017). Changes in food selectivity in children with autism spectrum disorder. J. Autism Dev. Disord..

